# Challenges and Accomplishments of Overseas‐Trained Nurses in Australian Aged Care: A Qualitative Study of Indian Nurses’ Experiences

**DOI:** 10.1155/jonm/6278608

**Published:** 2025-12-11

**Authors:** Bindu Joseph, Sheba Joseph, Robeena Emmanuel

**Affiliations:** ^1^ Institute of Health and Wellbeing, Nursing, Federation University, Berwick, Victoria, 3806, Australia, federation.edu.au

**Keywords:** Australia, experience, challenges, Indian nurses, nursing homes, overseas-trained nurses, residential aged care

## Abstract

Appropriate and adequate staffing is critical in Australian Residential aged care facilities (RACFs). The multicultural workforce in RACFs is constantly increasing to address workforce shortages and maintain the quality of care provided. Indian nurses comprise a significant proportion of the registered nurses (RNs) working in RACFs in Australia. As we work to enhance staffing levels in RACFs, it is equally essential to provide proper support for overseas‐trained nurses in their roles. This research aims to explore the experiences of RNs trained in India and working in or have worked in Australian RACFs, focusing on the factors that have influenced their work in these facilities. This study adopted a qualitative descriptive methodology. Fourteen Indian nurses (*n* = 14) who had completed their basic nursing qualifications in India and worked in the aged care sector in Australia participated in this study. The diversity of the participants was maintained in terms of years of experience and work location (metropolitan and regional). One‐on‐one semistructured interviews were conducted via a virtual platform (Teams or Zoom). The interviews were transcribed and coded descriptively. This study identified various vulnerabilities, challenges, supportive factors, and opportunities experienced by Indian nurses at the Australian RACFs. Five themes emerged from this study: “The journey through a challenging transition,” “psychological impact,” “rewarding experience,” “support system,” and “pathways to improve.” Working in RACFs is a rewarding experience despite the challenges associated with transition issues, workload, and cultural diversity. Specific experiences can even psychologically impact nurses working in aged care facilities. The availability of support personnel, orientation programs, and feedback opportunities positively impacts the effective transition and overall experience of Indian nurses in residential care settings.

## 1. Introduction and Background

Residential aged care facilities (RACFs) provide a supportive living environment for older people who require physical and psychological support in their daily lives. The number of people over 65 years using RACFs has increased by 3.1% over the last 5 years, from 172,000 on 30 June 2017 to 178,000 on 30 June 2022 [1]. As demand for residential aged care (RAC) services increases among older individuals, there is significant pressure on the health system to maintain the quality of care provided by these services. Based on the Royal Commission into Aged Care Quality and Safety, the Australian government has introduced various initiatives, including 24/7 registered nurse (RN) staffing and mandatory care minutes in RAC homes to maintain and enhance the quality of care [2]. All these factors have collectively led to an increased demand for nurses in RACFs. According to the Nurse Demand and Supply Study, there is a projected undersupply of 17,551 full‐time equivalent (FTE) nurses in the aged care sector by 2035 [2]. Foreign nurses significantly contribute to addressing these workforce deficiencies [3]. According to the 2020 Aged Care Workforce Census, 35% of the total RACF direct care workforce is from Culturally and Linguistically Diverse (CALD) backgrounds [4]. Of those workforces, 24% comprises nurses. Due to the shortage of nurses and the growing demand for nurses in RACFs, the multicultural workforce in Australian healthcare will continue to increase. Exploring the experiences of Indian nurses in Australian aged care is particularly significant due to several reasons. Indian nurses are Australia’s second‐largest group of overseas‐qualified nurses. [5]. Indian nurses often receive minimal formal training in geriatric or aged care [6], and many have little to no experience in aged care settings. Furthermore, culturally, strong family values enable them to care for the elderly with respect and compassion [7]. These cultural norms and hierarchical values could also affect how they convey and express their concerns, making them a vulnerable group [3, 8]. Additionally, nursing education in India often involves training in resource‐limited settings, fostering adaptability and resilience. These traits influence how Indian nurses practice and integrate compared with other CALD groups whose training contexts may differ significantly [3]. This lack of preparation, combined with cultural uniqueness, can impact their transition to Australian RACFs, where nurses are expected to manage the complex care needs of older adults, work within multidisciplinary teams, and navigate regulatory and clinical systems.

Previous research by Joseph et al. [3] explored the transition experiences of Indian nurses in Australian mental healthcare settings, identifying key challenges such as role ambiguity, isolation, and cultural challenges during their early integration into the Australian workforce. While that study provided an initial overview of transitional stressors, the current study extends this work by focusing specifically on Indian nurses in aged care settings. Other studies have also highlighted that the transition to a foreign healthcare setting is challenging for nurses with CALD backgrounds [9–11]. The experiences of nurses have a direct impact on care quality and job satisfaction [12–14]. Studies indicate that migrant nurses frequently encounter challenges related to cultural differences and workplace integration [3, 15–17]. Understanding the experiences of international nurses is key to addressing workforce challenges [18, 19]. Evidence suggests that fostering an inclusive work environment improves overall workforce effectiveness and satisfaction [20, 21]. Studies also suggest that tailored training and support programs are essential for overseas‐qualified nurses to effectively integrate and perform in new healthcare environments [22–25]. Insights from the proposed study can guide policy and practice improvements in aged care settings. Identifying the challenges experienced by Indian nurses can inform strategies to enhance their integration into the Australian healthcare system. Understanding the experiences of Indian nurses can help identify barriers to inclusion and highlight opportunities to better support culturally diverse staff, thereby contributing to more inclusive practices.

### 1.1. Conceptual Framework and Methods

This qualitative study used the theory of intersectionality as a framework. The in‐depth interviews capture the complex and contextual experiences of Indian nurses working in Australian aged care settings. The theory of intersectionality [26] examines how different aspects of a person’s identity, such as ethnicity, race, and cultural identity, intersect to create unique experiences. Indian nurses working in aged care have a unique ethnic identity, which may shape their experience within the Australian aged care system. Additionally, the experience of cultural adjustments, values, and beliefs can influence their interpretation of experiences in the aged care settings. All three researchers started their nursing careers in Australia as overseas nurses from India. They have firsthand experiences of different challenges that overseas nurses face.

Participants were recruited through aged care facilities across Australia. Researchers contacted facility managers via email to advertise the study. Additional participants were identified through snowball sampling, whereby existing participants referred others to the study.

This study received ethics approval from the 14 nurses who completed their basic nursing qualifications in India and are working or have worked in the aged care sector in Australia. Participation was voluntary; participants were provided with a plain‐language statement outlining the nature of the study, and informed consent was obtained from all participants before the interview.

Interviews were conducted via virtual platforms (Microsoft Teams or Zoom) and explored participants’ experiences in providing care to aged care residents, the challenges they faced, and suggestions for improvement. An interview guide was in place. Participants were asked to share background information, including their country of nursing qualification, total years of nursing experience, and duration of residence in Australia. They were invited to describe their experiences working as RNs in Australian aged care settings, highlighting aspects such as job satisfaction, challenges encountered, and the impact of their work on both mental and physical health, with specific examples. The interview also explored how aged care nursing affected their personal lives, the enjoyable elements of their work, and the types of support they received. Finally, participants were asked for their views on how support for Indian‐aged care staff, could be improved, and were allowed to share their additional comments.

The interviews were recorded, transcribed verbatim, and analyzed using thematic analysis. Themes were identified and coded, and relationships between themes were examined to understand participants’ experiences better. Thematic analysis was conducted using the six‐step reflexive thematic analysis approach outlined by Braun and Clarke. This included familiarization with the data, generation of initial codes, searching for themes, reviewing themes, defining and naming themes, and producing the report [27].

Credibility was ensured through member checking, where participants were invited to review the preliminary findings, and one participant did so. The reflexive journaling process, ongoing research team meetings, and the provision of rich descriptions to support transferability ensured validity and reflexivity.

Two researchers independently analyzed the content, and the information was organized into codes, subcategories, categories, and finally, into main themes. These steps ensure clarity, rigor, and optimize the accuracy of the data analysis process [28].

## 2. Results

A total of 14 participants were interviewed. Seventy‐nine per cent of the participants were females. Most participants had 5–10 years of experience in aged care in Australia, and six had completed postgraduate degrees in nursing. Four participants had less than 5 years of experience. Guided by the theory of intersectionality, the analysis revealed how participants’ experiences were shaped not only by their unique transition experience but also by the interplay of race, gender, language, and external supports.

Most of the participants were female (79%) and aged between 31 and 40 years (71.4%). Most held a Bachelor of Nursing (57%), with the remainder having postgraduate qualifications (43%). In terms of their experience in Australian aged care, half had 5–10 years, 29% had less than five years, and 21% had over 10 years of experience (Table 1). The demographics broadly reflect the workforce trends reported in national data [29], where nurses are primarily female, midcareer, and hold bachelor‐level qualifications.

**Table 1:** Participants’ characteristics.

Characteristics	*n*	%
*Age*		
20–30	2	14.3
31–40	10	71.4
41–50	2	14.3

*Gender*		
Female	11	79
Male	3	21
Prefer not to disclose	0	0

*Qualifications*		
Bachelor of Nursing	8	57
Postgraduate Qualification	6	43

*Years of Aged Care Experience in Australia*		
Less than 5	4	29
5–10	7	50
10 and above	3	21

### 2.1. Thematic Analysis of the Experience

The thematic analysis of the interview elicited five core themes (Table 2).

**Table 2:** Themes.

Themes
The journey through a challenging transition
Psychological impact
Rewarding experience
Support system
Pathways to improve

### 2.2. “The Journey Through a Challenging Transition”

Most participants described starting work in Australian aged care as a “big transition” for them, marked by challenges related to language, culture, and unfamiliar systems. Language barrier was frequently highlighted by the participants, affecting their ability to communicate with both residents and colleagues. One participant shared, *“My language was a big challenge. You know, three or six months does not make me a proficient English user, so I was struggling”* (Par 10). This was further compounded by cultural differences, lack of assertiveness, food habits, and people’s behavior, which required ongoing adjustment. This was reflected in the statement by another participant,

“*I found it hard to say no to many situations… even if it was stressing me a lot*” (Par 5). The challenge extended beyond interpersonal communication to navigating an unfamiliar healthcare system, policies, and computer‐based documentation. As one nurse explained, being new to the country made it particularly challenging*. “I was new to all the rules and regulations, the policies even”* (Par 13).

Further to this, participants discussed challenges associated with the leadership roles and responsibilities, as well as overall adaptation. They highlighted that most of the time, there is only one RN during the shifts, and they often had to take leadership roles, which in turn heightened their stress and workload. One participant’s comment on the responsibility and need to act as “*the go-to person*” (Par 8), especially during after‐hours, reflects the pressure placed on migrant nurses. Furthermore, the reluctance of team members to accept the newly migrated nurse as a team leader has contributed to further complexities associated with workplace dynamics. As one participant mentioned, *“You are a new person in that facility Sometimes, people don’t see you as a leader for that role”* (Par 8).

### 2.3. Psychological Impacts

The psychological toll of aged care work was evident, with many nurses reporting stress, fatigue, sleep disturbance, and emotional strain. The participants reflected on various psychological stress they experienced, including “*feel like get stressed if you’re tired, your brain doesn’t work”* (Par 9), and

*“I was mentally down in the initial times”* (Par 14). The psychological impact was influencing their physical health as one participant mentioned *“Some days, I used to cry and couldn′t sleep”* (Par 5) as well as affecting their life outside the workplace, as reflected by another participant *“I bring work home it took me a while to unwind”* (Par 1).

Participants also revealed feelings of “discrimination” based on their skin color and ethnicity, by residents and colleagues. Several nurses reported being looked down upon or treated unfairly due to their overseas background, with one stating, “people look down on you…even colleagues” (Par 10). Discriminatory behaviors from residents, including refusal of care from nurses with darker skin tones, further contributed to a sense of exclusion and marginalization. One participant shared that there were comments from residents like “…I don’t like that Indian nurse.” (Par 8). These highlight some of the unique pressures experienced by overseas‐qualified nurses.

### 2.4. Rewarding Experience

Despite the challenges, participants found working in aged care settings and caring for older people is a rewarding experience. *“I enjoy working with older people…It’s rewarding to feel the difference that we make as individuals trying to care for them, trying to help them, and the difference that brings in their life that stands out, and that’s quite rewarding”* (Par 4). Participants enjoyed working with the families. One participant stated, “*I enjoy it when their families recognize you. They know we have been, you know, following up or caring for their loved ones. It’s rewarding”* (Par1). Elders are highly respected in Indian culture, and participants were happy to contribute towards various aspects of their care. *“It is not only about the job, it is more about feeling part of the family and feeling like caring for a family member, incorporating my spiritual and moral values*” (Par. 4). The continuity of care associated with the aged care setting was also noted as a positive factor by the participants. “*The* enjoyable element is that you can see different people and how they go through the process of ageing.”

*For me, it’s I’m helping somebody for a long time”* (Par 13). This included end‐of‐life care. “*It’s rewarding. You get to know the resident from day one to their last day”* (Par 14). This connection and experience served as a motivational factor for migrant nurses working in aged care.

### 2.5. Support Systems

Participants highlighted various support systems they had during their initial time in aged care in Australia. Support from colleagues and management played a crucial role in facilitating adjustment and job satisfaction. Some participants mentioned that senior staff and managers provided guidance, orientation, and emotional support, as one said, “*My manager was very supportive, the manager herself took me to the town and showed me around these other areas*” (Par 7). They appreciated this support, which extended beyond their workplace. However, support varied across facilities, indicating inconsistencies that could affect new nurses’ experiences.

### 2.6. Pathways to Improve

Participants in the study shared their views on improving the experiences and transition of new migrant nurses in Australian aged care settings. Comprehensive orientation programs were repeatedly emphasized as essential, providing education on local systems, policies, and clinical expectations. As participants stated, “*A well-organized orientation program will be helpful”* (Par 9) and “*The most important thing is to give them proper education and training. A good orientation is very, very important”* (Par 5). Open communication and regular feedback opportunities were also recommended to ensure nurses feel heard and supported in their roles. For example, one participant reflected, *“Open communication is important. I wanted to give feedback on my experience and suggest a few things to support future staff”* (Par 14). Another participant suggested “*There should be face-to-face meetings with your manager allocated very month to discuss how you’re going, how things are improving, how you feel,* etc.” (Par 13). These recommendations offer practical avenues for improving workplace culture and migrant nurses’ well‐being.

## 3. Discussion

In this study, the theory of intersectionality was used as a conceptual lens to explore the experiences of Indian nurses working in Australian aged care settings. Their transition journeys and lived experiences were shaped not only by their professional roles as RNs but also by intersecting factors such as racial and ethnic identity, migrant status, language background, and gender. These intersecting identities influenced how Indian nurses perceived their challenges and the support available to them within aged care environments.

While Indian migrant nurses have been the focus of a substantial body of literature, limited research has specifically examined their experiences in aged care facilities. This study found that Indian nurses working in the Australian aged care system face challenges that stem from both their migration journey and the nature of the aged care work environment. The findings are presented under five key themes: “the journey through a challenging transition,” “psychological impact,” “rewarding experience,” “support system,” and “pathways to improve.”

Participants in this study reported that migration and beginning work in RACFs marked a significant transition, particularly due to language barriers, cultural differences, and unfamiliar systems and practices. These findings echoed a study conducted in New Zealand where Indian nurses experience several physical, social, and professional hardships as they transition to aged care facilities [11]. Other studies also highlighted the difficulty immigrant nurses face in engaging in casual conversations with residents, primarily due to limited exposure to local culture and informal language use [10, 30]. This unfamiliarity often led to misunderstandings [31]. Language barriers are consistently identified as a significant challenge, affecting communication not only with residents but also with colleagues and other staff [10, 32]. Participants in the study discussed that they face difficulties in standing up for themselves and being assertive in many situations. This was identified in other studies as well, where Indian nurses highlighted that verbalizing workplace concerns due to cultural norms and hierarchical respect inhibits assertive communication and reporting of issues [33]. These communication difficulties contributed to frustration for many nurses, who also felt that they were unable to provide the high‐quality nursing care they were capable of [12, 15]. It was also identified by nurses from non‐English speaking backgrounds that communication difficulties in English influence job satisfaction in Australian hospitals [14]. Similarly, Angus et al. [16] reported that cultural differences and communication barriers contributed to internal struggles among migrant nurses in New Zealand’s aged care sector. Discrimination in the workplace, particularly race based, was another key challenge highlighted in this study. Such experiences can negatively affect the well‐being of healthcare workers and impact the delivery of high‐quality care [20]. Participants described feeling disrespected or treated differently, echoing findings from studies that reported discrimination against migrant nurses in Australia, Germany, and the UK [34] and that immigrant nurses often feel less respected compared to their locally born counterparts [35]. These discriminatory and socioenvironmental factors can contribute to significant psychological effects, including stress, fatigue, and sleep disturbances, as described by several participants. Similar findings are noted in the literature, where migrant nurses experience high levels of stress, burnout, and job dissatisfaction [18, 36]. Many participants in this study raised concerns about deskilling and underutilization of their skills in aged care settings. Globally, internationally qualified CALD nurses in aged care are often underutilized, raising concerns about deskilling and limited opportunities for career development [37]. Experiences of discrimination [38, 39] and professional skepticism from colleagues regarding their capabilities [10] further compounded these impacts.

Despite these challenges, many participants also expressed a sense of satisfaction and fulfilment in their roles. Similar to the findings by Zanjani et al. [17], nurses in this study found working in Australia to be rewarding. Some were positively surprised by the kindness and friendliness of patients, as reported by internationally qualified nurses in New Zealand [40].

Participants shared that they enjoyed the autonomy in clinical decision‐making and leadership responsibilities, which required strong clinical knowledge and confidence. While some felt that their specialized skills were fading in the aged care setting, they chose to remain in the sector because they found value in their caregiving roles. This phenomenon of deskilling is not unique to Indian nurses and has been widely recognized among overseas‐trained professionals [31]. Nevertheless, many migrant care workers continue to take pride in their caregiving and appreciate the relational aspects of their work [41]. Studies indicate that Indian nurses hold strong religious and spiritual perspectives on ageing and end‐of‐life, framing caregiving as not only a professional role but also an act with moral significance [7]. This was reflected in the participants’ accounts, as they described bringing positive cultural values into their practice, demonstrating profound respect for older adults and integrating spirituality and cultural traditions into all aspects of care, including the provision of end‐of‐life care.

Participants highlighted the importance of support systems in easing their transition. Structured orientation programs and support from colleagues played a crucial role in helping them adapt. Supportive colleagues were especially valuable in helping overseas‐trained nurses integrate into the Australian healthcare context [25]. In addition to informal support, participants suggested more formal structures to improve the experience for migrant nurses. Echoing the findings of Smith et al. [34], they called for structured integration programs that include language support and country‐specific bridging initiatives to address organizational barriers. Cultural training and learning support during the transition period were seen as key to adapting smoothly to the new environment [23]. Ongoing education was also seen as beneficial, consistent with the experiences of internationally qualified nurses in New Zealand [42]. Raising awareness about these challenges and making systemic changes at the organizational level may be a sustainable path forward to better support the integration and retention of internationally qualified nurses in Australian RACFs.

## 4. Limitations

This study focused on Indian nurses within the aged care context in Australia. While the study is in‐depth, the findings may not be generalizable to other migrant nurse groups, other healthcare settings, or countries. Data were collected through online interviews, which offered flexibility; however, this method had some limitations. Nonverbal cues, such as body language or subtle emotional reactions, were challenging to observe, which may have affected the depth of interpretation.

## 5. Recommendations

Undoubtedly, there is a clear need for comprehensive orientation programs that include cultural training, system navigation, and regular performance feedback. Additionally, incorporating language support, including communication workshops tailored to healthcare settings and assertiveness training might help Indian nurses to feel more confident in voicing their concerns and advocating for residents. Mentorship is also an important strategy to empower these nurses. This can involve pairing overseas‐trained nurses with experienced, culturally aware mentors, including senior Indian nurses who have lived experience of cultural and professional transition. This peer guidance can ease adaptation, build confidence, and create a safe space for professional and emotional support. Addressing workplace discrimination and promoting leadership development can also support better integration and retention of overseas‐trained nurses in Australian aged care facilities. At the organizational level, employers should prioritize antidiscrimination policies and leadership development programs to enhance workforce integration and retention. Finally, connecting the Indian nurses with local Indian community organizations and peer support groups can provide them with cultural familiarity, social connection, and mental health support.

## 6. Conclusion

This study identified key challenges faced by Indian nurses working in Australian RACFs, including language and cultural differences, limited exposure to aged care, unfamiliar healthcare systems, and discrimination from colleagues and residents. While these issues are not unique to Indian nurses, their specific cultural and professional backgrounds, beliefs, and nursing training in India, as well as hierarchical concepts shape their experiences uniquely. Such differences further complicate their transition into Australia’s aged care work environments. Working in RACFs was rewarding for many of them, particularly in terms of autonomy, leadership, and utilizing their critical thinking skills. The findings of this study also indicated that these nurses felt more confident and comfortable as they gained more experience in Australian aged care settings. While there was variation among participants in terms of prior aged care exposure, nursing experience, and length of stay in Australia, the study primarily explored challenges as they were experienced during the transition period, regardless of background. The participants reported that increased familiarity with the Australian aged care system led to improved confidence, job satisfaction, and professional autonomy. Facilitating access to culturally relevant support networks, as recommended, may further enhance their transition and improve overall experience in the aged care workforce. [43]

## Conflicts of Interest

The authors declare no conflicts of interest.

## Funding

This research did not receive any funding.

## Data Availability

The data that support the findings of this study are available from the corresponding author upon reasonable request.
